# Up-to-Date Imaging and Diagnostic Techniques for Prostate Cancer: A Literature Review

**DOI:** 10.3390/diagnostics13132283

**Published:** 2023-07-05

**Authors:** Ming Zhu, Zhen Liang, Tianrui Feng, Zhipeng Mai, Shijie Jin, Liyi Wu, Huashan Zhou, Yuliang Chen, Weigang Yan

**Affiliations:** Department of Urology, Peking Union Medical College Hospital, Chinese Academy of Medical Sciences and Peking Union Medical College, Beijing 100730, China

**Keywords:** prostate cancer, diagnosis, imaging technique for diagnosis, biopsy, literature review

## Abstract

Prostate cancer (PCa) faces great challenges in early diagnosis, which often leads not only to unnecessary, invasive procedures, but to over-diagnosis and treatment as well, thus highlighting the need for modern PCa diagnostic techniques. The review aims to provide an up-to-date summary of chronologically existing diagnostic approaches for PCa, as well as their potential to improve clinically significant PCa (csPCa) diagnosis and to reduce the proliferation and monitoring of PCa. Our review demonstrates the primary outcomes of the most significant studies and makes comparisons across the diagnostic efficacies of different PCa tests. Since prostate biopsy, the current mainstream PCa diagnosis, is an invasive procedure with a high risk of post-biopsy complications, it is vital we dig out specific, sensitive, and accurate diagnostic approaches in PCa and conduct more studies with milestone findings and comparable sample sizes to validate and corroborate the findings.

## 1. Introduction

Prostate cancer (PCa) is one of the most prevalent diagnosed cancers globally with 1,276,106 new cases in 2018, and a prominent reason for tumor-related mortality in men [1,2]. The incidence increases with each decade of age, and therefore, 59% of men over 79 years of age have suffered from PCa [3]. Generally, most of the prostate cancers are indolent in nature, but others are invasive and are diagnosed either when already metastasized or rapidly spread beyond the prostate, resulting in morbidity and potential prostate cancer-specific mortality [4].

PCa mortality has lowered in the past decades, mainly due to the widespread application of preliminary exams [5]. Transrectal/transperineal prostate biopsy is the current mainstream prostate cancer diagnosis technique in most areas of the world, one which is conducted for a raised prostate specific antigen (PSA) value and/or abnormal findings on digital-rectal examination [6]. Established screening methods based on PSA level could significantly improve the rates of early diagnosis, and almost 90% of PCa can be localized clinically at the time of its diagnosis [7]. PSA screening is currently suggested for all males over 50 years, based on the European Society of Medical Oncology (ESMO) recommendations from 2020. Nevertheless, secreted by prostate epithelial cells, PSA is considered as an organ-specific rather than a cancer-specific marker. Because of its organ-specific nature, preoperative serum PSA has been considered as a predictor for tumor volume in PCa patients undergoing radical prostatectomy (RP). In recent decades, early detection of PCa and its clinical management have become debatable topics, because the current primary non-invasive prostate cancer screening methods have resulted in an unsatisfactory amount of unnecessary prostate biopsy cases. It is commonly well-accepted that clinically significant PCa or high-grade PCa (GS (Gleason score) ≥ 7) benefit the most from treatment, which includes either radiotherapy or radical surgery, and thus the risk of overdiagnosis and overtreatment of indolent PCa restricts PSA implementation at a population level [8,9]. Serum total PSA level could be abnormally increased due to benign prostatic hyperplasia, infection, etc. [10]. On the other hand, data from the Prostate Cancer Prevention Trial (PCPT) highlighted that 14.9% of PCa in men occurs with PSA levels lower than 4.0 ng/mL burden and Gleason scores of seven or higher [11]. Recent studies that have applied an exacting reference criterion have demonstrated that over half of men with clinically significant prostate cancer were incorrectly diagnosed when exposed to transrectal ultrasound (TRUS) biopsy [7]. Biopsies are an invasive procedure that has a high risk of post-biopsy complications, including per rectal bleeding, hematuria, and sepsis. Therefore, it is necessary to find a new and effective approach in order to diagnose clinically significant PCa (csPCa).

In this literature review, we discuss the indications and imaging techniques for prostate biopsy, summarize recent data concerning the diagnostic performance of several modern techniques, and compare the diagnostic and prognostic utility of prostate cancer tests.

## 2. Discussion

### 2.1. Conventional Imaging

For local lesions, ultrasound has the advantage of being non-ionizing and repeatable. The TRUS probe, which is in close proximity to the prostate gland, provides clearer images compared to computed tomography (CT) or magnetic resonance imaging (MRI), and has thus become a commonly utilized imaging diagnostic technique for prostate cancer. Currently, the ultrasound methods mainly include two- and three-dimensional TRUS, doppler ultrasound, ultrasound quantification, and acoustic radiation force impulse imaging. Two-dimensional (2D) ultrasound is a classic technique for prostate imaging, but it can only detect a small number of PCa cases (11~35%) [12]. Only 17~57% of the hypoechoic nodules displayed in the 2D ultrasound images are malignant [13], indicating poor sensitivity and specificity. However, due to the clear imaging and easy operation, it is still the preferred method for guiding systematic prostate biopsy in clinical practice. Three-dimensional (3D) ultrasound enables axial and sagittal imaging, and coronal and three-dimensional images are reconstructed by computer. Sedelaar et al. noted that, compared with 2D ultrasound, 3D ultrasound has increased sensitivity: 74% vs. 85%; but this sensitivity is associated with a decrease in specificity: 52% vs. 41% [14]. Mitterberger et al. discovered that the sensitivity and specificity of 3D ultrasound for extraprostatic lesions were 84% and 96%, respectively, and among the 16 patients, 14 were classified as T3b, indicating its role in clinical staging of PCa [15]. In recent years, several ultrasound quantification techniques have emerged for the diagnosis of prostate lesions, including the automated urologic diagnostic expert system (AUDEX), computer-aided transrectal ultrasound (C-TRUS) technology, and HistoScanning. Simmons et al. performed a comparative analysis of HistoScanning ultrasound results and pathology following RP for prostate cancer, indicating a sensitivity and specificity of 90% and 72%, respectively, for HistoScanning [16]. Doppler ultrasound enables the visualization of blood flow and its velocity in PCa, thereby reflecting the extent of PCa proliferation and its malignancy [17]. It encompasses two modes: color Doppler flow imaging (CDFI) and power Doppler ultrasonography (PDU). Zhao et al.’s study suggested a significant improvement in the cancer detection rate (CDR) of PDU compared to TRUS for patients with a PSA greater than 10 ng/mL [18]. The aforementioned techniques have achieved a certain success in visualizing prostate cancer. However, given the existence of interobserver bias, ultrasound is still mainly used as the guidance for prostate biopsy.

Regarding metastases, conventional imaging methods mainly include CT, bone scintigraphy (BS), and whole-body MRI (WB MRI). CT is of limited value in detecting intraprostatic lesion and local staging, but of use in metastases of lymph node or bone. CT or MRI nodal staging relies on assessment of lymph node morphological criteria such as size or shape [19]. The metastatic node may be of normal size, while the enlargement is possibly due to reactive hyperplasia caused by infection or inflammatory reaction. However, AUA guidelines still recommend CT for intermediate- to high-risk PCa patients, because of its easy accessibility and low cost. A radionuclide BS following the injection of a technetium-99m (^99^mTc) tracer is currently the standard and most widely used method of evaluating bone metastases of intermediate- to high-risk PCa [20,21]. A meta-analysis comparing choline-PET/CT, MRI, SPECT, and BS for diagnosing bone metastases showed a pooled sensitivity of 79% (95% CI: 73–83%), and a specificity of 82% (95% CI: 78–85%), respectively, at patient level for BS [22]. However, with the emergence of new techniques such as ^68^Ga-PSMA-PET/CT, it is expected that CT and BS are to be gradually phased out. Pasoglou et al. indicated that WB MRI showed higher sensitivity and specificity than did combined BS [23]. WB MRI provides high soft-tissue contrast and can display anatomical details with great precision, while avoiding ionizing radiation. It is noteworthy that existing research suggests that the sensitivity of WB MRI may be slightly lower than that of PET [24,25]. However, more data is needed, especially in evaluating the potential complementary roles of WB MRI and PET.

### 2.2. mpMRI

The hypoechoic lesions on TRUS are not specific to PCa; thus, the accuracy of TRUS in guiding prostate biopsy depends on the operator’s knowledge and experience [26,27]. Since MRI-guided prostate biopsy was first conducted by D’Amico in 2000 [28], it has been proven, through high-quality research, to detect more csPCa with fewer biopsy cores than does system biopsy [29]. The standard multiparametric magnetic resonance imaging (mpMRI) protocol is the combination of multiple planars, including T2-weighted imaging (T2WI), diffusion-weighted imaging (DWI), and dynamic contrast-enhanced (DCE) sequences [30]. mpMRI is assessed through the Prostate Imaging-Reporting and Data System (PI-RADS) and is currently revolutionizing the PCa diagnostic pathway [29]. Under the guidance of mpMRI, radiologists can detect, score, and stage lesions that may correspond to csPCa, the statuses of which can later be verified through histopathological grading [7]. Previous study has proved that mpMRI is a promising tool, with sensitivity ranged between 44% and 93% and specificity ranged between 38% and 94% [31]. Wide variations in sensitivity and specificity can be explained by different acquisition protocols, different thresholds being applied, different reference criteria used, biopsy inaccuracies, and the varied experience levels of the radiologists with PI-RADS v2. Multiple guidelines have recommended the routine application of mpMRI in biopsy-naïve patients with suspected PCa, and there is an ongoing implementation process in clinical practice [32].

Patients with a previous negative biopsy could also benefit from mpMRI as a method for omitting unnecessary repeat biopsy. Oishi et al. indicated that men with negative mpMRI, a previously negative biopsy, and a PSA density (PSAd) lower that 0.15 ng/mL/cc can safely omit rebiopsy [33]. The EAU/EANM/ESTRO/ESUR/SIOG guidelines strongly suggested conducting the mpMRI before repeat biopsy to select a target lesion in repeat biopsy settings [20]. All in all, 64% of urologists thought mpMRI was useful in detecting PCa for biopsy-naïve men, while 97% regarded it as valuable in men with a negative biopsy [34].

Nevertheless, significant interobserver variability remains to be a non-ignorable drawback for MRI [35], leading, in the literature, to overall heterogeneous findings for accuracy [36]. A multicenter, multi-reader study with six expert prostate radiologists detected moderate reproducibility for PIRADS version 2, but also found “considerable inter-reader variation” [37]. Even so, radiologists should have the requisite training, experience, and clinical volumes to routinely read prostate MRI images to maintain adequate expertise. In recent years, efforts have been devoted to exploring whether mpMRI could allow better evaluation of PCa aggressiveness, through both conventional statistics metrics [38,39] and higher-order texture features based on T2WI and apparent diffusion coefficient (ADC) maps [40,41,42]. Several important characteristics, including tumor grade and size, have been proved to affect conspicuity on mpMRI; tumor location and its association with mpMRI visibility remain undetermined facets of this area [43]. Therefore, it is necessary to investigate additional techniques for adequate management of the technique. 

Bi-parametric (bp) MRI (a combination of T2WI and DWI) has also been introduced as a substitute for mpMRI with gadolinium-enhanced sequences, reducing the costs and potential side effects of gadolinium-based contrast agents [30,44,45]. As of yet, multiple studies have shown that the application of bpMRI protocols would not significantly decrease PCa detection rates, and that it is comparable to mpMRI protocols [46,47]. Based on PI-RADS V2.1, the role of the DCE sequence is only helpful for score 3 lesions in the peripheral zone (PZ) [48]. Nevertheless, evidence on their diagnostic performance is insufficient, and potential limitations of abbreviated protocols (such as an increased number of equivalent findings) should be investigated. Besides, DCE-MRI is itself a fairly applicable strategy to show neovascularization, and one normally applied to acquire kinetic information of image intensity enhancement. Whether the role of DCE can be replaced by other parameters is a proposition that still requires high quality research for proof.

### 2.3. Combination with Biomarkers

When considering advancements in the MRI pathway, it is apparent that the overall detection rate for csPCa, specifically defined as ISUP Grade Group (GG) 2, is approximately 38–40% [7,29,49]. This rate may seem relatively low, primarily due to the considerably lower detection rate observed for PI-RADS 3 lesions (12% in the PRECISION trial) compared to PI-RADS 4/5 lesions (60% and 83%, respectively) [29]. Studies have indicated that forgoing biopsies in patients who exhibit no lesions on an MRI could potentially result in missing approximately 5–11% of all incidences of csPCa [50,51]. Conversely, conducting biopsies in all patients with equivocal MRI findings (PI-RADS 3 lesions considered positive by guidelines [52]), may lead to a csPCa diagnosis in approximately 3–50% of these patients [50,51,53,54]. PI-RADS 3 is considered critical for several reasons. Firstly, the detection rate for PI-RADS 3 lesions remains relatively low, leading to a higher likelihood of men undergoing unnecessary biopsies. Secondly, the interpretation of PI-RADS 3 lesions can vary depending on the experience of the radiologist, resulting in inconsistencies in the number of lesions categorized as PI-RADS 3. In such circumstances, laboratory diagnostic biomarkers can play a role complementary to that of MRI in the diagnosis of csPCa.

According to a study by Friesbie et al., the implementation of a PSAd threshold of ≥0.1 ng/mL/cc demonstrated an improvement in the detection of csPCa, by 7% for PI-RADS 3, 17% for PI-RADS 4, and 15% for PI-RADS 5, respectively, on a per-patient basis [55]. The IMRIE study retrospectively analyzed 2642 men and showed that incorporating the standard PSAd threshold of ≥0.15 ng/mL/cc into the MRI pathway resulted in increased sensitivity and negative predictive value (NPV) for GG ≥ 2 (ranging from 87.3% to 96.6% and from 87.5% to 90.6%, respectively). Moreover, the utilization of a PSAd of 0.12 ng/mL/mL further improved sensitivity and NPV [56]. Preliminary studies have indicated the potential benefits of incorporating PSAd into the decision-making process for repeat biopsy in cases of active surveillance or during follow-up for men with a negative targeted biopsy [57]. 

Besides PSAd, other advanced laboratory biomarkers, such as 4Kscore and the prostate health index (PHI), have been utilized in conjunction with mpMRI to further augment the detection of csPCa. The 4Kscore test encompasses the assessment of four kallikreins (total PSA (tPSA), free PSA (fPSA), intact PSA, and human kallikrein 2 (hK2)), in conjunction with age, findings from digital rectal examination (DRE), and a record of previous prostate biopsy. In a retrospective series conducted by Wagasker et al., a nomogram was proposed combining 4Kscore with mpMRI, one which demonstrated promising results with area under the curve (AUC) values of 0.84 for any PCa, 0.88 for csPCa, and 0.86 for ≥GG3 PCa [58]. PHI is a biomarker which combines tPSA, percentage fPSA, and −2proPSA. Nomograms developed by Siddiqui et al. incorporating serum biomarker data (PHI, %fPSA, or tPSA) and mpMRI along with other clinical variables have demonstrated high accuracy in both training cohorts and independent cohorts. In the independent validation cohort, a nomogram for ≥GG2 PCa using PHI as a biomarker resulted in a reduction of 39.1% unnecessary biopsies (143/366) while only missing 0.8% of csPCa (1/124), with a biopsy threshold of 20% probability of csPCa. Indeed, these biomarkers are readily available and do not involve further tests combined with imaging. However, it is worth noting that despite their potential, these biomarkers may still be underutilized in clinical practice, and further studies are necessary.

### 2.4. Fusion Targeted Biopsy

Recent trends and evidence advocate pre-biopsy MRI with selective targeting of suspected malignant lesions under the guidance of MRI/ultrasound (US) and a targeted biopsy (TB) approach for its advantage to elevate the detection rate of csPCa, with a reduction in the overdiagnosis of clinically insignificant cancers [7,29]. This strategy allows for lesion-directed mpMRI TB and optimal planning of a biopsy. Through this approach, men who had positive results on the mpMRI underwent an MRI-targeted biopsy with the application of real-time ultrasonographic guidance, a technique which could allow MRI targets to be visualized on the ultrasound [29]. Recent research has proved that mpMRI and mpMRI/TRUS fusion targeted biopsy have demonstrated good accuracy in the diagnosis of csPCa, especially for the cancer located at the anterior zone of the gland [59]. As the PRECISION study indicated, MRI-ultrasound (US) fusion biopsy significantly outperforms systematic biopsy regarding CDR: 38% vs. 26% with *p* = 0.005 [29]. Generally fusion prostate biopsy (FPB) can be carried out in the form of TB, which is based only on acquiring specimens from suspicious lesions, or in combined biopsy (CB), in which 10- to 12-core standard biopsy (SB) is performed in addition to TB [60]. During CB, as expected, the quantity of cores sampled per target and the total biopsy time are increased [61]. Thus, the current controversy is centered on the question of whether one should continue doing the systematic biopsy along with MRI guided biopsy. 

The findings of J.P. Radtke et al. prove that a combined TB+SB provides improved csPCa detection rates over either systematic or MRI-targeted biopsy or mpMRI alone, and that 97% of csPCa incidence has been detected by combined TB + SB [62]. However, this strategy is related to an increased quantity of biopsy cores, and frequently, with increased detection of indolent disease [63,64], and we are still far from safely selecting patients who might benefit from MRI-TB alone, relying on the combination of patient characteristics and mpMRI parameters. Therefore, the combination of MRI-TB and TRUS-guided biopsy (TRUS-Bx) should strongly be recommended as the best available approach for reducing the risk of csPCa misdiagnosis and the option offering the most reliable depiction of PCa multifocality.

To date, three approaches have been introduced: cognitive fusion TB (COG-TB), software-based fusion TB (FUS-TB), and in-bore or in-gantry TB (IB-TB). The first approach is “cognitive” targeting, where the physician conducting a transrectal US-guided biopsy reviews the MR imaging results before the procedure and applies this knowledge to identify the most suitable area for TB, as guided by US images [65]. The second approach involves superimposing the MRI images onto the TRUS images after paired landmarks are generated in both through MRI-TRUS fusion platforms. The third approach, in-bore MRI target biopsy (MRI-TB) is performed in the MRI suite through real-time MRI guidance [66]. 

COG-TB is a more cost-effective and accessible targeted biopsy strategy, especially for small institutions, or those without fusion software or equipment for MRI in-bore biopsy; however, primarily according to the operator’s tumor identification, COG-TB needs a higher level of experience and a more easily followed template to reduce operator variability, and there are insufficient data on the optimal template and predictors for the detection rate of COG-TB. It has been proven that, compared with computer fusion biopsy, cognitive fusion is characterized by a lower cancer confirmation rate for lesions located in the anterior zone or in the transition zone of the prostate [59,67,68,69]. Puech et al. compared COG-TB vs. FUS-TB and detected no difference in cancer detection rate [70]. While most research findings are derived from data from developed countries, there are a few studies from low- and middle-income countries that analyze the impact of COG-TB through mpMRI data on the detection of clinically significant cancer. At the same time, compared against software, further study is needed to pave the way for incorporating MRI-targeted COG-TB, at least until mpMRI fusion biopsy is more widely available.

Progress in the fields of information technology and artificial intelligence has resulted in the development of software platforms that support clinical diagnosis and decision-making through patient data from personalized medicine. The MRI-US fusion biopsy platforms have several advantages, including real-time overlay of the MRI and ultrasound images (with precision similar to the in-bore targeted biopsy), possibility of concurrent systematic sampling, and shorter duration of the procedure when compared with in-bore sampling [64,71]. Multiple versions of targeted biopsy software exist and are capable of conducting biopsies of suspicious regions on the prostate MP-MRI. Uncertainty about the effect of different software-based imaging processing techniques remains to be explored [72]. MRI-TRUS software fusion is also considered less expensive than would be an MRI-guided biopsy (MRGB), and it can be conducted in a shorter time [73]. 

The European Association of Urologists guidelines suggest that in biopsy-naïve patients with a suspect lesion, systematic TRUS-Bx should be followed by MRGB directed at the MRI-suspicious areas, while in patients with a persisting clinical suspicion of PCa after having gone through a systematic TRUS-Bx with prior negative results, only MRGB targeted to the lesion is recommended [74]. MRGB allows real-time control of the sampling correctness, potentially decreasing errors during the targeting process [75]. Nevertheless, in-bore biopsies fail to allow for concurrent systematic sampling because of time limitation; thus, lesions missed by the prebiopsy cannot be detected by mpMRI through this modality. Another shortcoming is the limited space within the MRI bore, thus limiting the range of motion of the physicians within the magnet. Additionally, the research sample for the in-bore technique is relatively small compared to other trials on mpMRI-TB. This is mainly caused by the fact that in-bore targeted biopsy is neither widely available nor widely applied.

### 2.5. PET/CT

The sensitivity of conventional imaging approach such as CT, MRI, or BS often fails to detect sites of relapse and/or metastasis [76]. Molecular imaging of prostate cancer has demonstrated good results and allows whole-body assessment of tumor biology. In recent decades, positron emission tomography (PET) techniques have arisen as an encouraging tool for PCa detection, tumor staging, and treatment planning, including metastatic castration-resistant prostate adenocarcinoma [77,78]. PET combined with CT or MRI can help to localize suspicious lesions in the prostate gland through prostate-specific radiotracers (i.e., ^18^F-fluorodeoxyglucose (FDG), Fluorine-18-labeled sodium fluoride (^18^F-NaF), choline—labelled with either ^18^F and ^11^C, ^18^F-Fluciclovine, ^18^F-16b-fluoro-5a-dihydrotestosterone (^18^F-FDHT), and prostate specific membrane antigen (PSMA) ligands labelled with ^68^Ga or ^18^F), thus providing a valuable tool for the diagnosis of cancer, and for the initial staging of the disease [79]. Different radiotracers for PET imaging have been explored in recent years and each tracer has different uptake features. With the occurrence of nuclear tracers, PET could map changes in function and metabolism rather than in anatomy only [80].

^18^F-FDG PET/CT in the initial staging of PCa is controversial [81,82], since the uptake of FDG is generally low in PCa cells compared with other malignant cells, which causes difficulty in separating malignant from benign tissue [83,84]. The NCCN guidelines recommend against ^18^F-FDG PET/CT in the initial staging of PCa, but instead advise evaluating biochemical recurrence (BCR) or metastasis [85]. However, H. Jadvar has insisted that FDG PET/CT may be of use in the diagnosis and staging of primary tumors with GS > 7, given the tendency to display high FDG uptake [81].

The uptake of ^18^F-NaF does not directly allow for the visualization of the presence of tumor cells. Instead, it reflects the increased blood flow, osteoblastic activity, and bone remodeling that are associated with osseous metastases. In comparison to BS, ^18^F-NaF demonstrates increased bone uptake and a more rapid clearance from soft tissues (owing to minimal serum protein binding). This leads to enhanced bone-to-background contrast and shorter examination duration [86]. At present, there is insufficient evidence to endorse, for clinical benefit, the routine use of ^18^F-NaF PET/CT instead of BS. Additionally, the bone-only detection capability of ^18^F-NaF PET/CT presents a limitation, making it a less attractive option in the current age of developing targeted tracers for molecular imaging of prostate cancer which can simultaneously detect extra-skeletal disease.

Radiolabeled choline is an extensively studied tracer in the restaging of prostate cancer in BCR [87]. Choline is an essential element of phospholipids in the cellular wall, and the elevated uptake of choline leads to increased metabolism of the cell membrane components of malignant tumors [88]. Up to now, the EAU has suggested PET imaging with choline derivatives for BCR after radical prostatectomy with a PSA serum level ≥ 1 ng/mL [89]. 

^18^F-Fluciclovine PET/CT has also shown promise in the detection of recurrent prostate cancer, with significant impact on subsequent treatment planning, and it has been approved by the Food and Drug Administration (FDA) and the European Commission for patients with elevated PSAs for BCR following prior treatment [90]. The FDA approval was due to its high diagnostic performance and the histologically confirmed data in patients with BCR, with a 68% detection rate on a per-patient basis, a 62% positive predictive value on a per-lesion basis (greater than 90% for extra prostatic disease), and a 70% specificity level for lesions [91]. Fluorine-18-labeled fluciclovine PET/CT has also been demonstrated to be effective for evaluating distant metastases in recurrent PCa [91,92]. Detectability of F-18 fluciclovine PET/CT is generally increased as the PSA level is elevated, and it is more sensitive at PSA level > 1 ng/mL with rapid PSA kinetics [93]. As for PSA levels less than or equal to 1 ng/mL, detection rates range only from 21.0% to 46.4% [94,95,96]. Correspondingly, up to now, there has still been no absolute lower-level cutoff for PSA value that has been explored as an indication for ^18^F-fluciclovine PET/CT which would provide guidance to referring physicians (urologists, radiation oncologists, and medical oncologists) in determining which patients might benefit most from imaging. 

In the context of advanced CRPC resistant to initial conventional ADT, ^18^F-FDHT holds significant promise. This tracer specifically targets the androgen receptor (AR), which, along with its natural ligands, testosterone and 5a-dihydrotestosterone, plays a crucial role in male sexual differentiation. However, the use of ^18^F-FDHT PET/CT is currently limited to investigational research purposes, and it has not yet been approved for clinical routine use. Preliminary studies investigating the use of ^18^F-FDHT PET/CT in patients with CRPC have demonstrated safety, feasibility, accurate detection of lesions, and a correlation with survival outcomes [97,98].

To date, no single imaging approach has demonstrated optimal diagnostic performance in the evaluation of metastatic lymph nodes. Studies with the PET tracers ^18^F-choline and ^11^C-choline have indicated similarly high specificities, but low sensitivities have ranged from 40% to 50% [99]. Because of the low-spatial resolution of PET imaging (approximately 5 mm in clinical scanners), choline PET/CT has been found to be unable to diagnose sub-centimeter node metastasis. Beheshti et al. demonstrated a sensitivity of 66% and specificity of 96% for lymph nodes larger than 5 mm when applying ^18^F-choline PET/CT [100]. It is important to clarify the advantages and disadvantages of ^18^F-choline PET/CT use for imaging tests of PCa patients. 

### 2.6. PSMA PET

Prostate-specific membrane antigen (PSMA) is a type II transmembrane glycoprotein receptor with folate hydrolase activity and glutamate carboxypeptidase activity. PSMA expression is elevated significantly in higher-grade prostate tumor cells and other solid cancers, including advanced salivary gland cancer, glioblastoma, thyroid cancer, hepatocellular carcinoma, and clear cell renal carcinoma [100]. PSMA can easily penetrate tissues and diffuse within solid tumor lesions and thus reflect the metastasis situation [101,102,103]. Compared with mpMRI, a distinct advantage of ^68^Ga-PSMA PET scans is that PSMA is overexpressed up to 1000-fold in prostate malignancies compared to benign tissues, which theoretically makes PSMA PET scans relatively specific for malignant transformation compared to mpMRI [104].

Fendler et al. assessed 21 patients for the accuracy of ^68^Ga-PSMA- PET/CT in localizing a tumor in the prostate and surrounding tissue and detected a significantly higher SUVmax in histopathologically positive segments (11.8 ± 7.6) when compared with negative segments (4.9 ± 2.9; *p* < 0.001) [105]. ^68^Ga-PSMA PET/CT is also a prevalently applied modality in patients with BCR, as it has been proved to be superior to conventional diagnostic imaging for locating recurrent disease. Although a BCR after RP is defined by a PSA value > 0.2 ng/mL based on guidance [106], and PSMA PET/CT imaging is suggested for patients with a PSA value > 0.2 ng/mL according to the EAU guidelines [107], the patient group with pre-scan PSA values < 0.2 ng/mL could also be included in determining the effects on the outcome. 

PSMA-PET/CT has shown its accuracy in restaging patients in either the local, nodal, or metastatic setting. ^68^Ga-PSMA has demonstrated greater diagnostic accuracy and a competitive advantage over ^18^F-fluciclovine in detecting recurrences at PSA levels down to less than 0.5 ng/mL [108,109]. A systematic review including 4790 patients demonstrated patient-based PCa BCR detection rates of 33% and 45% at PSA levels < 0.2 and 0.2–0.49 ng/mL, respectively [110]. Guevelou et al. summarized the potential impact of restaging according to PSMA-PET/CT changes in the management of recurrent prostate cancer after RP [111].

Currently, there is also convincing evidence on the re-staging efficacy of Ga-68-PSMA-11-PET in men with non-metastatic castration-resistant prostate cancer (CRPC). Fendler et al. performed a multicenter, retrospective study in 200 CRPC patients with serum PSA levels over 2 ng/mL, and/or a Gleason score ≥ 8, in whom conventional imaging showed the absence of metastasis; the Ga-68-PSMA-11-PET/CT demonstrated positive findings in 98% of these patients. In addition, in 55% of the patients who suffered disease recurrence, metastases were found in the extra-pelvic lymph nodes (39%), bone (24%), and visceral organs (6%) [112]. ^68^Ga-PSMA PET/CT imaging has also demonstrated impressive early results, to the extent that some consider it to be the reference standard for the detection of lymphatic metastases [113,114]. Metastatic lymph nodes can appear with non-specific characteristics on MRI; thus, traditional imaging modality will always fail to detect lymphatic metastases [115]. The higher diagnostic accuracy of ^68^Ga-PSMA-11 PET/CT over mpMRI for pelvic lymph node staging prior to radical prostatectomy in patients with intermediate- to high-risk PCa were confirmed according to the most recent evidence, with a sensitivity of 71% and a specificity of 92% [116].

However, up to now, there has still been no recommendation on the routine application of ^68^Ga-PSMA-11 PET/CT imaging for the initial staging of PCa according to the current EAU-EANM-ESTRO-ESUR-SIOG Guidelines [20]. The EAU guidelines still rate their recommendation for PSMA-PET/CT in the setting of BCR after radical prostatectomy as “weak”, and fluciclovine-PET/CT is endorsed where PSMA-PET/CT is not available. Because most of the studies exploring PCa primary detection and initial staging before definitive therapy are retrospective design and composed of intermediate to high-risk PCa patients, these tumors are more likely to overexpress PSMA; thus, a potential bias and overestimated accuracy is possible [117,118,119,120]. Some studies have proved the limitation of ^68^Ga-PSMA PET in detecting low- and intermediate-risk PCa, one which is caused by the low prevalence of extra-prostatic lesions [121]. As expected, some small lesions are missed due to the limited spatial resolution of PET and the presence of background activity in the urinary tract. Additionally, ^68^Ga-PSMA-11 is metabolized through the urinary system and thus accumulates in the urinary bladder. 

At the standard imaging time 100 min after injection, this could result in obscuration of local recurrence in the prostatic fossa by overlaying activity in the urinary bladder. Radiopharmaceutical ^18^F-PSMA-1007 is a novel PSMA-based radiopharmaceutical that has multiple advantages compared with ^68^Ga-PSMA-11 [122]. ^18^F-PSMA-1007 is not excreted from the kidneys in the first few hours after injection, which is potentially beneficial in the detection of local recurrences [123]. In addition, the end-point positron energy of [^18^F]-F is much lower than that of [^68^Ga]-Ga (0.65 vs. 1.90 MeV), which could decrease the positron range in tissue and thus improve spatial resolution [124]. Nevertheless, Rauscher et al. have indicated that, despite the aforementioned superiority of [^18^F]-PSMA-1007 over [^68^Ga]-PSMA-11, they also noticed a high incidence of unspecific bone uptake—which could lead to over-staging—in a significant number of patients [125].

### 2.7. PET-Target

mpMRI based fusion biopsy often misses some foci located at the transition and central zones, and the specificity of mpMRI for detecting PCa also decreases its diagnostic efficacy as a triage tool for biopsy. PET is also a useful tool for improving the accuracy of imaging-guided biopsy in PCa, one which mainly includes three approaches: PSMA PET/CT-TRUS software-assisted fusion biopsy, transgluteal PSMA PET/CT-targeted prostate biopsy, and cognitive PSMA PET/CT-TRUS-targeted prostate biopsy. Up to now, PET/CT-biopsy guidance has recommended its use only in patients with previous negative biopsy [126], but it is considered a promising potential tool for future diagnosis [127]. The process of PSMA PET/CT-TRUS software-assisted fusion-targeted prostate biopsy includes selection of suspected PCa lesions by PSMA PET/CT, incorporation of the PSMA PET/CT data into real-time TRUS by an imaging fusion system, and then the acquisition of biopsy samples, as guided through the fused TRUS imaging in real-time. Transgluteal PSMA PET/CT-targeted prostate biopsy means identifying PCa lesions through PSMA PET/CT imaging and then taking two to four biopsy specimens by single-puncture percutaneous transgluteal method under real-time CT guidance, as initially introduced by Zhang et al. in May 2020 [128]. When applied to cognitive PSMA PET/CT-TRUS-targeted prostate biopsy, the surgeon needs to view the location of a target lesion identified by PSMA PET/CT imaging and then translates the suspected PCa lesion sites to be targeted by TRUS-guided biopsy through various mental processes, such as memory, measurement calculation, three-dimensional spatial reasoning, and pattern recognition. 

Simopoulos et al. first introduced a successful case of ^68^Ga-PSMA PET/CT and MRI/ultrasound-guided prostate biopsy [129]. Subsequently, Westenfelder et al. successfully detected csPCa (GS 4 + 3) through ^68^Ga-PSMA PET/MR-plus-ultrasound guided biopsy in a patient with a previous negative prostate biopsy and an MRI result [130]. Limited available evidence has shown the superior performance of PSMA-PET in comparison with mpMRI for lesion characterization and intra-prostatic staging [127,131,132]. Caracciolo et al. highlighted that PSMA-PET/MRI has a high accuracy for detecting csPCa, and is a promising tool for the selection of patients with suspicion of PCa and preceding negative biopsy or contraindications to MRI [133]. Ferraro et al. demonstrated that ^68^Ga-PSMA-11 PET/MRI-guided biopsy had a high accuracy for detecting csPCa with patient-based sensitivity, specificity, negative and positive predictive value, and accuracy of 96%, 81%, 93%, 89%, and 90%, respectively [134]. These preliminary results indicated that PSMA PET could be a useful tool in identifying and defining malignant lesions before prostate biopsy. Fendler et al. highlighted that lesions with SUVmax > 6.5 for PCa diagnosis would lead to a sensitivity of 67% and specificity of 92% when performing target biopsy [105]. SUVmax reflects the tumor expression of PSMA, with higher grade tumors (Gleason score > 7) usually relating to a much higher SUVmax value, varying from 16 to 21, as compared with intermediate and lower grade tumors corresponding to SUVmax values of 8.2–8.8 and 5.9–9.6, respectively [135,136]. 

In the study of R. Kumar et al., postprocedural complications were reported in five of fifty-six (9%) participants and were minor (i.e., hematuria, hematospermia, and gluteal pain), with no participant suffering from a postprocedural infection [137]. The transgluteal modality could decrease the risk of infection and the need for multiple prostatic capsule biopsy. ^68^Ga-PSMA PET/CT use in this context could significantly reduce unnecessary biopsies and related complications. Nevertheless, literature on PSMA-PET/CT-guided biopsy remains sparse. Few studies have made a direct comparison between outcome of mpMRI-guided biopsy and PSMA-PET/CT-guided biopsy for the detection of localized PCa. Besides, all the image tracers up to now have been labeled with ^68^Ga, and thus the value of ^18^F-PSMA PET/CT-TB remains to be investigated. More clinical studies applying PET/MRI-guided biopsy are recommended, and these may have better performance due to the superior spatial resolution provided by MRI. PET-guided biopsy could then be used when mpMRI is contraindicated or equivocal, or when an MRI-guided biopsy is negative for cancer despite high clinical suspicion.

The combination of mpMRI and PSMA-PET could offer complementary information, and the simultaneous use of both imaging approaches offers significantly augmented sensitivity and specificity for intraprostatic tumor mass delineation, and thereby generates an innovative imaging modality capable of overcoming the pitfalls of traditional imaging, and, potentially, helping clinicians in the management of PCa [138]. In addition, improved detection accuracy could help patient selection for salvage therapy and assist in guiding individualized treatments. ^18^F-FDG PET/ MRI could offer the anatomical imaging benefits of MRI over CT and the molecular imaging of ^18^F-FDG PET, without radiation or CT beam-hardening imaging artifacts. Furthermore, PSMA-PET/MRI enables whole-body staging at a lower radiation dose and the guidance of prostate biopsies in a single hospital visit, which is superior to the conduction of both a pre-biopsy mpMRI and a staging PSMA PET/CT.

### 2.8. PET/MRI

Multiple experience-based reports of PET/MRI have now been published. The simultaneous application of PET and mpMRI offers metabolic, structural, and functional imaging information regarding PCa status in a whole-body single-session test, along with better soft tissue contrast and reduced radiation exposure in comparison to PET/CT [139].

Eiber et al. demonstrated an overall detection rate of 84% in 75 patients with median PSA levels of 2.6 ng/mL through ^11^C-choline PET/MRI in recurrent PCa (range 0.2–88 ng/mL), and thus indicated a significant advantage for ^68^Ga-PSMA-PET/MRI over PET/CT and mpMRI modality alone for detecting malignant intraprostatic foci [117,140]. Hope et al. conducted a meta-analysis of ^68^Ga-PSMA-11 PET accuracy for the detection of PCa, with a sensitivity and specificity of 74% and 96% [141]. 

^68^Ga-PSMA-11 PET/MRI has demonstrated higher sensitivity and specificity than have either mpMRI or ^68^Ga-PSMA-11 PET imaging performed separately for detecting intraprostatic tumors and pelvic lymph nodes, especially for patients with extremely low levels of PSA (<0.5 ng/mL). Kranzbühler et al. published a retrospective study and reported a detection rate of 65% in patients with PSA values between 0.2 and 0.5 ng/mL [142]. In comparison, mpMRI has a reported sensitivity of 61% and specificity of 58.7% for the detection of local recurrence [143]. K.M. Selnæs et al. suggested that the quantity of equivocal findings was decreased when MR images were assessed combined with PET uptake [144]. PSMA-PET/MRI showed a patient-based accuracy of 90% for detecting csPCa, according to a prospective single-center study, with a sensitivity of 96% and specificity of 81% [134]. This is higher than the conventional mpMRI accuracy indicated in most studies applying template biopsy as a reference standard, including the PROMIS trial, which demonstrated sensitivity and specificity of 93% and 41%, respectively [7]. 

Very few studies so far have made a direct comparison between PSMA-PET/CT and PET/MRI for biopsy guidance. All of the literature up to now has been based on tests performed with patients with intermediate- to high-risk disease, which is not the patient population in which increased detection sensitivity is required. Thereby, further work in the active surveillance population is still needed. Domachevsky et al. compared ^68^Ga-PSMA PET/MRI with same-day late ^68^Ga-PSMA PET/CT according to lesion detectability in a mixed group of patients for initial staging of high-risk PCa patients (n = 13) and suspected recurrence (n = 8) and obtained a comparable agreement between the two imaging approaches [145]. I. Jambor et al. indicated that the use of neither PET/MRI nor mpMRI was not able to detect pelvic lymph node metastases smaller than 8 mm [146].

However, up to now, very limited data has been available as to its role in clinical setting, mainly owing to the high cost of PET/MRI. Besides, two aspects still need further investigation. First, MRI-positive lesions may show unapparent or low uptake in PET images, which may lead to misdiagnosis of some MRI-negative lesions as positive, if we regard all apparent uptake lesions as positive in PET images. Second, the cut-off value varies across different medical centers. Therefore, each study has been performed under different execution standards.

### 2.9. Radiomics

In recent years, radiomics has been explored as a means of adding value to diagnostic pathways and patient management. Radiomics is the transformation of medical images into high-dimension mineable data through the extraction of quantitative features, with the ultimate goal of applying these features to glean valuable diagnostic or prognostic information that can guide clinical decision making [147]. Radiomics provides a non-invasive and low-cost automated technique for the evaluation of tumor properties according to MR images. Thus, radiomics could offer additional data that are often not visible to the naked eye. Multiple approaches for csPCa segmentation or classification through deep-learning networks or radiomics methods have been reported in the literature [148,149,150]. One of the essential steps in radiomics is the obtaining of prospective protocol-based good-quality images. Multiple previous studies have already demonstrated that a radiomics-based machine learning method could help not only for the quantification of imaging results, but also has the potential to identify pathological findings without visible abnormalities. Another benefit is that radiomics examines whole tumors, as opposed to biopsy schedules, which tend to sampling errors caused by intra-tumoral heterogeneity [151]. Through radiomics analysis, noninvasive MR-based techniques could predict PCa aggressiveness prior to biopsy or surgery. Recent studies have investigated the value of radiomics according to MRI in the differentiation of PCa from benign prostate tissue [152]. The ability of MRI to visualize the whole tumor volume, in conjunction with ongoing attempts to standardize image acquisition parameters, offers the potential to explore the ability of quantitative image-derived features, or radiomics, to accelerate the development of accurate and reproducible predictors of disease progression on AS. Ginsburg et al. considered ADC radiomics indicators to be highly relevant for their transition zone classifier [153]. A quantitative radiomics method named the morphology, asymmetry, physiology, and size (MAPS) feature model reached an accuracy of 87% for prostate cancer detection [154]. S.J. Hectors et al. also demonstrated the additional value of MRI texture analysis for the identification of csPCa in PI-RADS 3 lesions, with a resulting sensitivity of 75% [155]. Min et al. and Zhang et al. have also assessed the outcomes of an mpMRI-based radiomics signature for csPCa diagnosis, with impressive results [156,157]. Multiple previous studies have applied radiomics analysis to the automation of PCa diagnosis and risk stratification [158]. 

Recently, M.C.F. Cysouw et al. indicated in a preliminary report the additional and potential value of PET-derived radiomics features through a machine-learning extraction method in a sample of patients with high-risk PCa [159].

However, in practice, one of the biggest problems of radiomics is generation [147]. The data on radiomics learning methods for the evaluation of the predictive or prognostic value of PSMA-PET are still unconvincing. The role of prostate MRI radiomics, from the whole prostate gland (WG) and/or from MRI-based suspicious lesions, remains the subject of controversy. This is mainly because the majority of the radiomics studies validated their methods through splitting their original dataset into training and validation subsets, while only a few studies have conducted a validation via an external set [158,160]. Radiomics analyses could probably help in treatment optimization, but this should be examined in large patient populations first. 

### 2.10. Cost-Effectiveness

The overdiagnosis of PCa leading to overtreatment, as well as the potential underdiagnosis of csPCa, represent inefficient utilizations of limited healthcare resources. The potential advantages of diagnostics may be evident in men whose prostate cancer is likely to progress. However, other men can be subjected to unnecessary tests and treatments, resulting in both economic costs to healthcare resources and harm to patients. Hence, determining whether the potential benefits of diagnostics outweigh the costs and harms necessitates a formal comparison of the costs and consequences of different courses of action through an economic evaluation. Reports have shown that a strategy using MRI may be cost-effective compared with systematic TRUS-Bx, not only in patients with prior negative TRUS-Bx [36], but also in biopsy-naïve populations [161]. Faria et al. conducted a cost-effectiveness study utilizing data from the PROMIS study, which demonstrated that the utilization of mpMRI followed by up to two targeted TRUS-Bx procedures was a cost-effective strategy for the early detection of PCa [162]. PSMA PET/CT is expected to incur higher costs compared to CT+BS when it comes to the primary staging of PCa. However, it is deemed cost-effective, particularly in newly diagnosed patients with intermediate- to high-risk PCa, as it helps to avoid unnecessary treatments [163]. The cost-effectiveness analysis conducted by Subramanian et al. indicates that PSMA PET/CT should be regarded as a viable alternative to both ^18^F-Fluciclovine PET/CT and standard imaging modalities for prostate cancer staging [164]. Regarding the BCR of PCa, both PSMA PET/CT and PSMA PET/MRI have been considered cost-effective approaches. Gordon et al. have demonstrated that the utilization of PSMA PET/MRI as a cost-effective alternative to the standard CT and BS staging scans for BCR can help to prevent unnecessary or ineffective local therapies and guide increasingly precise and targeted treatments [165]. However, according to the findings of Parikh et al., for PSMA PET/CT to no longer be considered cost-effective in PCa patients with BCR, its price would need to be set at or exceed $14,004 [166]. The evidence suggests that PSMA PET may, in the future, become an indispensable tool in the diagnosis and management of prostate cancer. However, currently, there is a lack of comparative cost-effectiveness studies between PSMA PET/CT and mpMRI. This may be due to the predominant focus of PSMA PET/CT cost-effectiveness research on lymph node metastasis and BCR, while studies on mpMRI’s cost-effectiveness tend to concentrate on intraprostatic lesions. Simultaneously, the issue of whether diagnostics for PCa are cost-effective in a particular country remains unclear. Therefore, any recommendations provided to decision-makers should take into consideration the current country-specific data.

## 3. Conclusions

The diagnostic techniques for prostate cancer develop with time. Since prostate biopsy is an invasive procedure which may cause post-biopsy complications, it is essential to discover non-invasive and increasingly accurate diagnostic approaches.

In this literature review, we have summarized the up-to-date existing techniques. (Table 1) For intraprostatic lesions, while conventional imaging techniques such as US and CT play their roles, mpMRI and various types of targeted biopsies are becoming increasingly important. Considering the csPCa detection rate, it is still recommended to utilize mpMRI for individuals who are biopsy-naïve or who require repeated biopsy. Simultaneously, the role of biomarkers in conjunction with mpMRI, as proposed in recent years, cannot be overlooked. For metastatic lesions and BCR, BS is the most accessible and widely employed method. Emerging PET diagnostic methods such as PSMA-PET and PET/MRI demonstrate promising diagnostic efficacy. However, due to cost constraints and lack of experimental data, their widespread application remains limited in the short term. Similarly, with the progress of artificial intelligence and machine learning, radiomics will play an increasingly significant role in future diagnostics.

Regarding the current state of affairs, the combined employment of MRI-TB and TRUS-Bx has been widely adopted for biopsy purposes to reduce misdiagnosis, and BS is widely used for screening metastases outside the prostate. In the future, we eagerly anticipate the emergence of novel imaging diagnostics that may gradually replace them. 

## Figures and Tables

**Table 1:** Summaries of the up-to-date imaging and diagnostic techniques for prostate cancer.

Methods	Studies	Year	Total Patients	Characters	Usage
2D TRUS	Beemsterboer, P.M., et al. [12]	1999	8600	Clear imaging and easy operation; poor SE and SP.	Preferred method for guiding SB
CT	——	——	——	Use in metastases of lymph node and bone; easy accessibility and low cost.	AUA recommendations for intermediate- to high-risk PCa.
BS	——	——	——	For metastases: SE 79% (95% CI: 73–83%), SP 82% (95% CI: 78–85%) [22].	The standard and most-used method for bone metastases of intermediate- to high-risk PCa.
mpMRI	Ukimura, O., et al. [27] Kasivisvanathan, V., et al. [29] Oishi, M., et al. [33]	2015–2019	762	SE ranged between 44% and 93%, SP ranged between 38% and 94% [31]; interobserver variability.	Routine application in biopsy-naïve patients; conduction before repeat biopsy to select target lesions.
mpMRI TB + SB	Ahdoot, M., et al. [60] Radtke, J.P., et al. [62]	2017–2020	2223	Detection rate of 97% for csPCa, superior to mpMRI (85%), TB (78%), and SB (88%) [62]; increase of the quantity of cores and total biopsy time.	Recommended as the best available approach to reduce the csPCa misdiagnosis.
PET/CT (^18^ F-Fluciclovine)	Bach-Gansmo, T., et al. [91] Andriole, G.L., et al. [92] England, J.R., et al. [94] Nanni, C., et al. [95] Odewole, O.A., et al. [96]	2015–2019	940	For patients with BCR, 68% detection rate, 62% PPV, 70% SP [91].	FDA and European Commission approval in patients with elevated PSA for BCR; no lower-level cutoff for PSA explored as an indication.
PSMA PET	Liu, C., et al. [101] Fendler, W.P., et al. [105] Hoffmann, M.A., et al. [107] Calais, J., et al. [108] Fendler, W.P., et al. [112] Jilg, C.A., et al. [113] Schmidt-Hegemann, N.S., et al. [114] Giesel, F.L., et al. [115] Hofman, M.S., et al. [119] Klingenberg, S., et al. [120] Cytawa, W., et al. [121] Donato, P., et al. [131]	2016–2022	1986	Patient-based PCa BCR detection rates of 33% and 45% at PSA < 0.2 and 0.2–0.49 ng/mL [110]; for pelvic lymph node staging prior to RP in intermediate- to high-risk PCa, SE 71%, SP 92% [116].	For patients with a >0.2 ng/mL PSA according to EAU guidelines; no recommendations for initial staging.
PET-target	Donato, P., et al. [127] Zhang, L.L., et al. [128] Ferraro, D.A., et al. [134] Kumar, R., et al. [137]	2020–2022	384	Detecting csPCa with patient-based SE 96%, SP 81%, NPV 93%, PPV 89%, and accuracy 90% [134]; decrease in unnecessary biopsies and complications.	Only recommended in patients with previous negative biopsy; a promising tool in the future diagnosis.
PET/MRI	Eiber, M., et al. [117] Park, S.Y., et al. [118] Eiber, M., et al. [140] Kranzbühler, B., et al. [142] Jambor, I., et al. [146]	2016–2020	253	SE 74%, SP 96% [141]; higher accuracy, especially for PSA < 0.5 ng/mL.	Limitations as to the cost and lack of data.

SE = sensitivity; SP = specificity; PPV = positive predictive value; NPV = negative predictive value; PCa = prostate cancer; csPCa = clinically significant prostate cancer; BCR = biochemical recurrence; PSA = prostate specific antigen; RP= radical prostatectomy; 2D TRUS = two-dimensional transrectal ultrasound; CT = computed tomography; BS = bone scintigraphy; mpMRI = multiparametric magnetic resonance imaging; TB = targeted biopsy; SB = systematic biopsy; PET/CT = positron emission tomography computed tomography; PSMA = prostate-specific membrane antigen; PET/MRI = positron emission tomography magnetic resonance imaging.

## Data Availability

No new data were created or analyzed in this study. Data sharing is not applicable to this article.
